# Synbiotic approaches to human health and well‐being

**DOI:** 10.1111/1751-7915.12789

**Published:** 2017-08-03

**Authors:** Thomas Gurry

**Affiliations:** ^1^ MIT Center for Microbiome Informatics and Therapeutics Cambridge MA 02139 USA; ^2^ Department of Biological Engineering Massachusetts Institute of Technology Cambridge MA 02139 USA; ^3^ The Broad Institute of MIT and Harvard Cambridge MA 02142 USA

## Abstract

Synbiotics refer to combinations of probiotics and prebiotics that act synergistically to confer health benefits to the host. As a therapeutic strategy, they provide a gentle yet powerful method for modulating the composition and metabolic output of the human gut microbiota. In the context of achieving the UN Sustainable Development Goals, synbiotics have the potential to act as cost‐effective prophylactic measures against a variety of human ailments, ranging from infant diarrhoea to metabolic and inflammatory diseases in adults, by maintaining commensal microbial communities and metabolic networks that are conducive to human health.

It is becoming increasingly clear that bacteria inhabiting the human gastrointestinal tract can play an important role in maintaining human health and that certain organisms, known as probiotics, can confer health benefits to the host when ingested. Certain food components have also been identified as being prebiotic, meaning that they assist in the growth and activity of probiotic or beneficial, commensal organisms. Synbiotics refer to synergistic combinations of prebiotics and probiotics (Pandey *et al*., 2015). As a therapeutic strategy, they have the potential to improve human health in a variety of different clinical indications. In particular, they hold great promise in addressing two targets in Goal 3 of the UN Sustainable Development Goals (SDGs): the prevention of neonatal mortality under the age of 5, as well as the prevention of premature mortality from non‐communicable diseases and promotion of mental health and well‐being.

The modulation of health by beneficial bacteria is thought to primarily occur through two mechanisms: the competitive exclusion of pathogens, and/or the secretion of metabolites that can impact host physiology. The success of faecal microbiota transplantation (FMT) in treating patients suffering from recurring *Clostridium difficile* infections has demonstrated the power of a healthy microbiota's ability to collectively ward off an infection, and though such a scenario has not yet been demonstrated in humans, one can anticipate treating a patient with particular commensal probiotic strains and their associated prebiotics to out‐compete pathogens that occupy similar niches in the gut. However, in the short‐term, it is likely that synbiotic therapies will be targeted towards the production of specific metabolites of clinical interest (Fig. 1).

**Figure 1:**
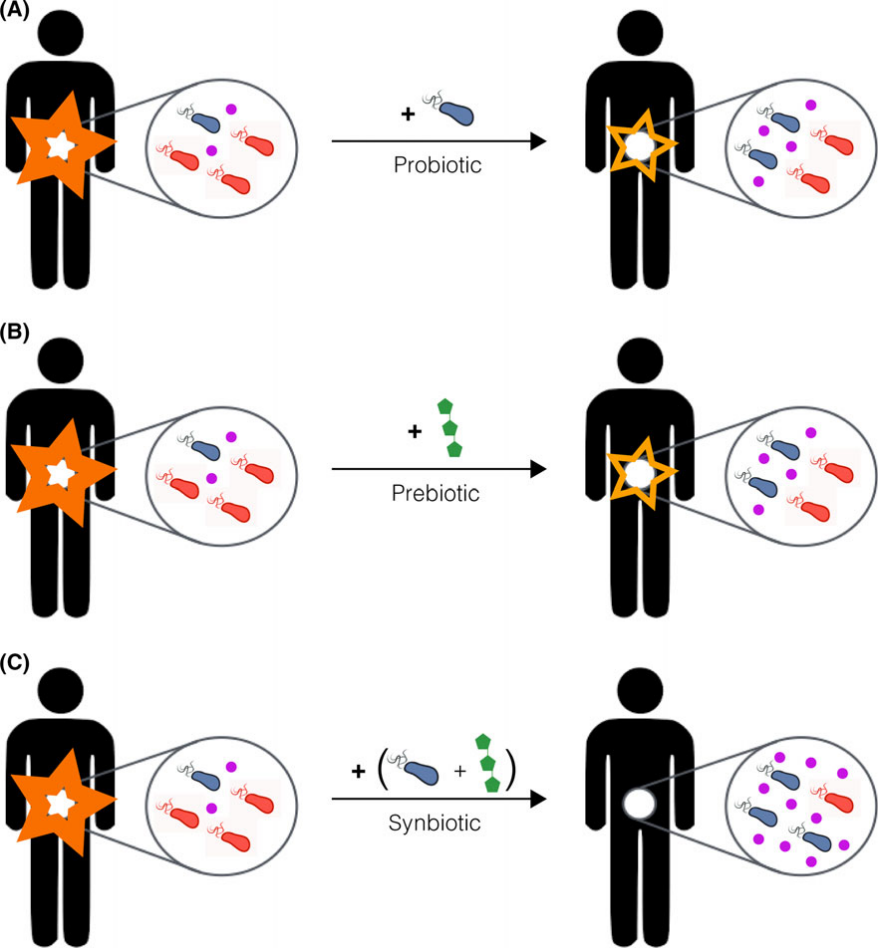
Schematic illustrating the synergistic effect on a subject's gut microbiota of administering a lactic acid‐producing probiotic (A) and an associated prebiotic (B) as a synbiotic formulation (C). In this example, the synbiotic aims to boost the production of lactic acid, depicted in purple, in order to reduce intestinal inflammation.

Current synbiotic approaches have focused on the most well‐characterised probiotics, which belong to the genera *Bifidobacterium* and *Lactobacillus*. These genera ferment human indigestible sugars such as fructooligosaccharides (FOS; Kaplan and Hutkins, 2000), so their synbiotic administration with FOS aims to multiply their relative abundance in the gut. The health effects of these probiotics are thought to result from the production of lactic acid from fermentation, which has been shown to act on the host's physiology in a number ways, including reduction in pro‐inflammatory cytokine secretion from TLR‐activated macrophages and dendritic cells (Iraporda *et al*., 2015), and reduction of the superoxide radical burden to intestinal enterocytes (Kahlert *et al*., 2016). Thus, the prebiotic also serves as a substrate for the generation of the physiologically active metabolite or precursor (in this case, lactic acid). This is reminiscent of the production of specific oligosaccharides in human breastmilk, which are indigestible to humans but provide fermentation substrates for lactic acid‐producing bacteria present in infant guts (e.g. *Bifidobacterium infantis*; Ward *et al*., 2006). These bacteria serve important functions in regulating neonatal health, resistance to infection and protection against infant diarrhoea prior to switching to a diet consisting entirely of solid foods (Morrow *et al*., 2004). Insights such as these can inform strategies aiming to reduce mortality due to diarrhoeal disease, the third leading cause of death worldwide in children under the age of 5 according to a 2013 estimate (Liu *et al*., 2015). For example, one could modify infant formula delivered to regions of the world in which the diarrhoeal burden and acquisition of early infancy pathogens are highest, by incorporating synbiotic formulations of these infant‐associated probiotics combined with synthetic milk oligosaccharide analogues. In the future, these formulations could be improved by including other organisms and their associated prebiotics as well. For example, it was recently shown that consortia of the strictly anaerobic, spore‐forming *Clostridia* provide colonisation resistance against pathogens such as *Salmonella* in infant mice (Kim *et al*., 2017).

Similar strategies can be extended into adulthood and to other conditions. As far as non‐communicable diseases are concerned, synbiotics offer promise in two main categories of human ailments: diseases related to inflammation, and diseases related to metabolism. An important component of a healthy gut, with implications to both of these categories, is the production of the Short Chain Fatty Acids (SCFAs) acetate, propionate and butyrate from the bacterial fermentation of dietary fibres in the colon (Ríos‐Covián *et al*., 2016). In particular, butyrate has been found to play a critical role in the modulation of intestinal inflammation (Furusawa *et al*., 2013), acting as a histone deacetylase inhibitor to modify gene expression in cells of the immune system (Boffa *et al*., 1978; Sealy and Chalkley, 1978). Indeed, depletion of butyrate producers has been associated with Inflammatory Bowel Disease (IBD) (Frank *et al*., 2007). Thus, synbiotic therapies combining butyrate‐producing probiotics with their fermentation substrate of choice are excellent candidates for the reduction of gastrointestinal inflammation‐associated disease burden worldwide. While diseases such as IBD do not have particularly high rates of associated mortality (Card *et al*., 2003), they dramatically impact afflicted individuals’ well‐being during periods of active disease, and constitute a significant economic and public health burden on the population. Moreover, they are increasing in incidence of emerging economies (Burisch *et al*., 2013), and so are important public health externalities to consider as these economies continue to develop. In a more speculative but equally promising direction, the epigenetic activity of butyrate and its diffusion into the host's blood stream suggest that it can, in principle, affect gene expression in non‐intestinal locations as well. For instance, intraperitoneal administration of butyrate to rats resulted in changes to hippocampal gene expression and apparent antidepressant effects as measured by behavioural tests (Yamawaki *et al*., 2012). Thus, although much work remains to demonstrate their viability in these contexts, synbiotics may play important roles in addressing depression and anxiety‐related disorders as well.

The gut microbiome has also been implicated in the aetiology of metabolic diseases. For instance, propionate and butyrate activate intestinal gluconeogenesis, which is believed to be responsible for the benefits of fibre‐enriched diets to glucose and energy homoeostasis (De Vadder *et al*., 2014). Increases in microbial acetate production and turnover, on the other hand, have been shown to activate glucose‐stimulated insulin secretion, which can lead to insulin resistance and subsequent obesity (Perry *et al*., 2016). Thus, modifying a patient's existing microbial SCFA production profile (e.g. by boosting the microbiota's net conversion of acetate to butyrate) may play an important role in addressing the increasing global incidence of metabolic diseases such as obesity and type 2 Diabetes. Additionally, promising microbial therapeutics could involve *Akkermansia muciniphila*, a gut commensal species that has been negatively associated with metabolic disorder (Derrien *et al*., 2017) and positively associated with healthier metabolic status in obese humans (Dao *et al*., 2016). Its abundance was found to increase in mice fed a diet enriched in fish oil (Caesar *et al*., 2015), suggesting a potential synbiotic strategy combining fish oil and *A. muciniphila* strains. Such synbiotic approaches may act as an important component in the reduction of the global incidence of metabolic disease, which in certain regions of the world is reaching epidemic proportions (Yoon *et al*., 2006). These diseases have strong associations with socio‐economic status (SES): in developed countries, they tend to be associated with lower SES, while in developing countries, they tend to be associated with higher SES (Wang, 2001). Successful application of synbiotic therapeutics, therefore, offers potential solutions to mitigate the increasing burden they place on lower socio‐economic groups as emerging economies transition to developed status.

Designing novel synbiotic strategies will depend on a molecular‐level understanding of the physiological effects of the microbially derived metabolome, and on detailed knowledge of the ecological forces and cross‐feeding relationships holding bacterial communities together in the gut. For instance, certain bacterial species (e.g. *Eubacterium hallii* and *Anaerostipes caccae*) utilise lactic acid as a substrate for butyrate production and therefore depend on the presence of lactic acid‐producing bacteria such as *Bifidobacterium adolescentis* in the culture medium to obtain butyrate as a metabolic output (Belenguer *et al*., 2006). To construct a butyrate‐producing synbiotic out of a probiotic like *Eubacterium hallii*, a minimal consortium might, therefore, consist of a lactic acid producer, an oligosaccharide (e.g. FOS) that would be fermented into lactic acid, and a lactate‐utilising butyrate producer. In addition, both probiotics may require their own growth‐promoting prebiotics to facilitate engraftment into the subject's microbiota. Thus, it is likely that synbiotics will often require more than simple pairs of a probiotic and an associated prebiotic, resulting in a complex and iterative discovery process. It is also worth noting that a large fraction of the gut microbiota can produce endospores, which allows them to lie dormant when environmental conditions are unfavourable for growth (Browne *et al*., 2016). The conditions for sporulation and germination are therefore critical variables requiring further study to make efficacious use of endospore‐forming probiotics. However, this also provides an opportunity for the design of endospore‐based synbiotics that only germinate in the gut. Such formulations would be much more resilient to variable or unreliable storage conditions prior to being administered to a patient, a property of particular relevance to remote or underdeveloped regions of the world.

The anticipated quantitative contributions of synbiotic therapeutics to addressing the UN SDGs are difficult to assess. In the examples discussed herein, there exist alternate strategies that synbiotics will likely complement rather than supplant. For example, reducing the burden of metabolic diseases such as obesity cannot be expected to occur from synbiotics alone; it is likely to also depend heavily on the promotion of healthy lifestyles, through public health measures ranging from improving dietary habits to promoting physical activity in the general population. Similarly, the treatment of severe diarrhoeal or inflammatory diseases will likely continue to involve non‐microbial therapies and surgical interventions (e.g. anti‐TNFα biologics and colonic resection in the case of IBD). Instead, the true impact of synbiotics may come from their use as prophylactic measures against these diseases. For example, addressing infant diarrhoeal diseases through synbiotic supplementation of infant formula aims to prevent their acquisition rather than to treat existing cases. As such, it is likely that synbiotics will play an important role in addressing the UN SDGs through disease prevention and maintenance of health rather than treatment of acute conditions.

## Conflict of interest

None declared.
